# Supplementary material for: When to stop: Understanding the landscape of extreme-duration cardiopulmonary resuscitation practices among pediatricians in Sudan
Source: PLoS One. 2025 Aug 1;20(8):e0328704. doi: 10.1371/journal.pone.0328704 (PMC12316234; doi:10.1371/journal.pone.0328704)
Supplement: S1 File — (PDF) [file pone.0328704.s001.pdf]

Thematic analysis of the open-ended question

The question: Please share any comments, insights, or experiences you have regarding prolonged CPR in pediatric patients with respiratory arrest and preserved pulse or heartbeat. This could include your thoughts on decision-making processes, ethical considerations, impacts on the healthcare team, or any other aspect you find significant. You can answer in English or Arabic.

Responses (each one participant's response could be divided into more than one theme):

	Participant's response (or part thereof)	Theme **(may appear a little different in wording from the final themes)**
1.	**The biggest problem now is the lack of oxygen, which makes pulmonary resuscitation ineffective in the first place...and we lose many patients.**	Shortage/lack of essential medicine and equipment to undertake prolonged CPR.
2.	**Training medical personnel on cardiopulmonary resuscitation**	Need for more/continuous training on CPR.
3.	**Availability of mechanical ventilator is a big challenge.**	Need for mechanical ventilator (unavailability makes prolonged CPR futile).
4.	**Providing specialized teams for resuscitation in all government hospitals in particular.**	Need for a dedicated resuscitation team in each hospital.
5.	**Providing life-saving medications in every ward**	Shortage/lack of essential medicines and equipment to undertake prolonged CPR.
6.	**Providing at least an mechanical ventilator on every floor**	Need for mechanical ventilator (unavailability makes prolonged CPR futile).
7.	**Protocol of CPR**	Critical need for developing clear protocol/policy for (when to stop) prolonged CPR/BMV (or when to continue prolonged CPR in the absence of MV).
8.	**Without a mechanical ventilator and a long period of non-breathing, it becomes difficult for resuscitation to be successful.**	Need for mechanical ventilator (unavailability makes prolonged CPR futile).
9.	**During my experience there's no recovery and the need for mechanical ventilator which is not available is adding more to the decrease benefits of CPR.**	Prolonged CPR/BMV is futile.
		Need for mechanical ventilator (unavailability makes prolonged CPR futile).
		Post-resuscitation critical care support makes prolonged CPR futile.

**10. Every doctor should learn Pediatric advanced life support (PALS).**	Need for more/continuous training on CPR.
**11. To provide hospitals with equipment for PALS and mechanical ventilators,**	Need for mechanical ventilator (unavailability makes prolonged CPR futile).
	Shortage/lack of essential medicines and equipment to undertake prolonged CPR.
**12. Enhance issuing protocols.**	Critical need for developing clear protocol/policy for (when to stop) prolonged CPR/BMV (or when to continue prolonged CPR in the absence of MV).
**13. Training for personnel.**	Need for more/continuous training on CPR.
**14. Establish a clear protocol (regarding when to stop CPR).**	Critical need for developing clear protocol/policy for (when to stop) prolonged CPR/BMV (or when to continue prolonged CPR in the absence of MV).
**15. I think that the inadequate pulse and breathing delivery within the 1st 20 to 30 mins of cardiac arrest are the most prognostic factors to the patient's outcome.**	Other
**16. My advice is to make sure that all healthcare providers are able to deliver efficient CPR.**	Need for more/continuous training on CPR.
**17. We need the consultant to participate in making the decision to stop CPR to ensure that you have not been unfair to the patient and that there is complete courage in making the decision.**	Difficult decision-making around prolonged CPR.
**18. The capabilities are not available in Sudan, including ventilator and CPAP.**	Need for mechanical ventilator (unavailability makes prolonged CPR futile).
**19. There should be a clear protocol that we follow, such that we take a specific period of time and even if the heartbeat continues, we stop. Consultants must talk to the family and explain to them.**	Critical need for developing clear protocol/policy for (when to stop) prolonged CPR/BMV (or when to continue prolonged CPR in the absence of MV).
**20. Because we would literally be standing in the arrested patient) performing CPR/bag-mask-valve ventilation) all day long, and the rest of the patients would be wasted (neglected).**	Need for a dedicated resuscitation team in each hospital.
	Prolonged CPR causes diversion of care from other patients.
**21. Consultants are rarely decisive about stopping CPR.**	Difficult decision-making around prolonged CPR.

**22. Lack of training of CPR provider leads to ineffective CPR.**	Need for more/continuous training on CPR.
**23. No clear guidelines regarding when to stop CPR.**	Critical need for developing clear protocol/policy for (when to stop) prolonged CPR/BMV (or when to continue prolonged CPR in the absence of MV).
**24. No practical benefit of prolonged CPR beyond 30 minutes if there's no mechanical ventilatory support facilities and ICU access.**	Need for mechanical ventilator (unavailability makes prolonged CPR futile).
	Post-resuscitation critical care support makes prolonged CPR futile.
**25. Good protocol should be available to make the decision of stopping CPR to face any ethical or legal consequences.**	Critical need for developing clear protocol/policy for (when to stop) prolonged CPR/BMV (or when to continue prolonged CPR in the absence of MV).
**26. Develop a protocol for such cases.**	Critical need for developing clear protocol/policy for (when to stop) prolonged CPR/BMV (or when to continue prolonged CPR in the absence of MV).
**27. We want a clear and detailed protocol for such cases.**	Critical need for developing clear protocol/policy for (when to stop) prolonged CPR/BMV (or when to continue prolonged CPR in the absence of MV).
**28. Direct contact numbers with pediatric emergency specialists that will be available 24 hours a day to respond in complex cases.**	Other
**29. We need trained CPR teams in every hospital available on every duty.**	Need for a dedicated resuscitation team in each hospital.
**30. We need to improve our hospital and staff about the important of CPR in pediatric patients in most hospitals in Sudan.**	Other
**31. Ethically and emotionally, you cannot stop CPR while the patient's heart is still beating.**	Emotional toll/moral burden on doctor if CPR is stopped while heart is still beating.
**32. A feeling of guilt that if you had continued the resuscitation process, he could have returned to life and not been the cause of a mother losing her child.**	Emotional toll/moral burden on doctor if CPR is stopped while heart is still beating.
**33. Mechanical ventilator very important.**	Need for mechanical ventilator (unavailability makes prolonged CPR futile).

**34. For me, there is no benefit from prolonged cardiopulmonary resuscitation for children, as it is stressful for the medical staff, especially since its termination depends on the decision of the specialist, who usually refuses for a period exceeding 12 hours for fear of legal problems.**	Prolonged CPR/BMV is futile.
	Difficult decision-making around prolonged CPR.
**35. Develop a clear protocol of CPR.**	Critical need for developing clear protocol/policy for (when to stop) prolonged CPR/BMV (or when to continue prolonged CPR in the absence of MV).
**36. CPR courses before and during the rotations.**	Need for more/continuous training on CPR.
**37. We need to learn how to do teamwork properly.**	Need for more/continuous training on CPR.
**38. Must provide mechanical ventilators and facilities.**	Need for mechanical ventilator (unavailability makes prolonged CPR futile).
**39. There should be a team available for CPR.**	Need for a dedicated resuscitation team in each hospital.
**40. The hospitals that do not contain mechanical ventilators make the act of CPR less valuable and useless, especially prolonged CPR.**	Need for mechanical ventilator (unavailability makes prolonged CPR futile).
**41. Develop a clear protocol for these cases, taking into account the capabilities of Sudanese hospitals in the event that mechanical ventilators are available after cardiopulmonary resuscitation and in the event that they are not available.**	Critical need for developing clear protocol/policy for (when to stop) prolonged CPR/BMV (or when to continue prolonged CPR in the absence of MV).
	Need for mechanical ventilator (unavailability makes prolonged CPR futile).
**42. Taking into account the number of staff in the hospital and the hospital capacity.**	Shortage/lack of human resources to undertake prolonged CPR.
**43. If every hospital prepared with MV devices this will make the outcome of CPR more favorable.**	Need for mechanical ventilator (unavailability makes prolonged CPR futile).
**44. Set a policy to stop CPR.**	Critical need for developing clear protocol/policy for (when to stop) prolonged CPR/BMV (or when to continue prolonged CPR in the absence of MV).
**45. YOU have to make protocol about CPR.**	Critical need for developing clear protocol/policy for (when to stop) prolonged CPR/BMV (or when to

	continue prolonged CPR in the absence of MV).
**46. We have no clear Protocol about stopping CPR.**	Critical need for developing clear protocol/policy for (when to stop) prolonged CPR/BMV (or when to continue prolonged CPR in the absence of MV).
**47. A protocol regarding "CPR termination and initiation in specific cases" is needed.**	Critical need for developing clear protocol/policy for (when to stop) prolonged CPR/BMV (or when to continue prolonged CPR in the absence of MV).
**48. We need supportive equipment like mechanical ventilation.**	Need for mechanical ventilator (unavailability makes prolonged CPR futile).
**49. If there is no ventilator in hospital or near place, I think CPR is of no benefits.**	Need for mechanical ventilator (unavailability makes prolonged CPR futile).
**50. Prolonged bag and mask ventilation after successful CPR due to lack of ventilation and proper post resuscitation care, patient arrested again, and CPR conducted again but unfortunately, we lost the patient.**	Need for mechanical ventilator (unavailability makes prolonged CPR futile).
	Post-resuscitation critical care support makes prolonged CPR futile.
**51. In our practice we conduct CPR properly in order to save the patient which if started early (cpr), the patient is lucky but then we are stuck in searching ICU bed for the patient and MV in order to complete the process with post resuscitation care. And we do our best with what we get.**	Need for mechanical ventilator (unavailability makes prolonged CPR futile).
	Post-resuscitation critical care support makes prolonged CPR futile.
**52. The presence of more intensive care is important to reduce the burden on doctors.**	Post-resuscitation critical care support makes prolonged CPR futile.
**53. We strongly need clear protocols for CPR and prolonged CPR.**	Critical need for developing clear protocol/policy for (when to stop) prolonged CPR/BMV (or when to continue prolonged CPR in the absence of MV).
**54. Lack of mechanical ventilatory support facilities is the main issue that must be encountered.**	Need for mechanical ventilator (unavailability makes prolonged CPR futile).

**55. I hope there is a clear protocol regarding the issue of prolonged resuscitation.**	Critical need for developing clear protocol/policy for (when to stop) prolonged CPR/BMV (or when to continue prolonged CPR in the absence of MV).
**56. The best outcomes of CPR depend almost always on the early intervention, the right decision and clear protocol.**	Critical need for developing clear protocol/policy for (when to stop) prolonged CPR/BMV (or when to continue prolonged CPR in the absence of MV).
**57. Establishing clear protocols is the solution.**	Critical need for developing clear protocol/policy for (when to stop) prolonged CPR/BMV (or when to continue prolonged CPR in the absence of MV).
**58. PICU unit at any hospital.**	Post-resuscitation critical care support makes prolonged CPR futile.
**59. Proper CPR is Teamwork.**	Other
**60. Need clearer protocol.**	Critical need for developing clear protocol/policy for (when to stop) prolonged CPR/BMV (or when to continue prolonged CPR in the absence of MV).
**61. More training needed to evaluate brain stem death signs.**	Need for more/continuous training on CPR.
**62. The need for a clear protocol for cases.**	Critical need for developing clear protocol/policy for (when to stop) prolonged CPR/BMV (or when to continue prolonged CPR in the absence of MV).
**63. Mechanical ventilator must be in any hospital.**	Need for mechanical ventilator (unavailability makes prolonged CPR futile).
**64. Some pediatric patients with respiratory arrest and preserved pulse can return to spontaneous respiration if do good face and mask ventilation for long time.**	Other
**65. Poor outcome.**	Prolonged CPR/BMV is futile.
**66. To share a protocol that suit every situation.**	Critical need for developing clear protocol/policy for (when to stop) prolonged CPR/BMV (or when to continue prolonged CPR in the absence of MV).
**67. I think when there are signs of brain death, we need to stop even if the heart is beating. Because in our poor resources it is not logical to continue with**	Prolonged CPR/BMV is futile.

**bagging "for long hours", taking turns every half an hour.**	
**68. prepare an ICU unit with ventilators.**	Post-resuscitation critical care support makes prolonged CPR futile.
	Need for mechanical ventilator (unavailability makes prolonged CPR futile).
**69. Prolonged CPR is dependent on the patient's condition and the time and facilities.**	Decisions around prolonged CPR should be individualized.
**70. Adopting a clear protocol regarding these cases, both religiously and medically.**	Critical need for developing clear protocol/policy for (when to stop) prolonged CPR/BMV (or when to continue prolonged CPR in the absence of MV).
	Accommodating the religious and legal aspect of the issue in policy/decision making.
**71. In my opinion, one of the most important reasons that leads to prolonged resuscitation is the absence of a clear protocol regarding when to stop prolonged cardiopulmonary resuscitation.**	Critical need for developing clear protocol/policy for (when to stop) prolonged CPR/BMV (or when to continue prolonged CPR in the absence of MV).
**72. Regular training for resuscitation program for medical staff.**	Need for more/continuous training on CPR.
**73. Clear protocol and guidelines.**	Critical need for developing clear protocol/policy for (when to stop) prolonged CPR/BMV (or when to continue prolonged CPR in the absence of MV).
**74. Focusing on pediatric intensive care specialization.**	Other
**75. We need recent guidelines for when to stop CPR.**	Critical need for developing clear protocol/policy for (when to stop) prolonged CPR/BMV (or when to continue prolonged CPR in the absence of MV).
**76. MUST be protected from law and religious responsibility.**	Accommodating the religious and legal aspect of the issue in policy/decision making.
**77. This is one of the most difficult clinical decisions. Both clinical staff and care givers should share in the decision-making.**	Difficult decision-making around prolonged CPR.
**78. In my opinion, to stop CPR is dependent on the outcome of patients illness and its prognosis in the future.**	Decisions around prolonged CPR should be individualized.

**79. There are a lot of services not available at our hospital for CPR.**	Shortage/lack of essential medicines and equipment to undertake prolonged CPR.
**80. Let them lay in peace.**	Prolonged CPR is futile
**81. Lack of medical personnel to carry out resuscitation and cover the rest of the work.**	Shortage/lack of human resources to undertake prolonged CPR.
**82. Lack of resources is one of the barriers to continue CPR as finding emergency trolly with ambulance, adrenaline, IV lines.**	Shortage/lack of essential medicines and equipment/resources to undertake prolonged CPR.
**83. Well trained team.**	Need for a dedicated resuscitation team in each hospital.
	Need for more/continuous training on CPR.
**84. The decision-making process should be based according to individual cases depending on each patient prognostic factors, for example if I have a syndromic patient or cerebral palsy or any congenital malformation the decision to stop resuscitation will be easier than a previously well patient.**	Decisions around prolonged CPR should be individualized.
**85. The lack of a clear protocol in these cases, and I believe that an opinion or Fatwa must be taken from scholars and religious scholars because in many cases this is a fundamental concern in the decision to stop resuscitation.**	Critical need for developing clear protocol/policy for (when to stop) prolonged CPR/BMV (or when to continue prolonged CPR in the absence of MV).
	Accommodating the religious and legal aspect of the issue in policy/decision making.
**86. There must be a clear protocol regarding the continuation of CPR in the absence of a ventilator.**	Critical need for developing clear protocol/policy for (when to stop) prolonged CPR/BMV (or when to continue prolonged CPR in the absence of MV).
**87. Any pediatric hospital should have PICU, mechanical Ventilation.**	Post-resuscitation critical care support makes prolonged CPR futile.
	Need for mechanical ventilator (unavailability makes prolonged CPR futile).
**88. Clear CPR and DNR policy.**	Critical need for developing clear protocol/policy for (when to stop) prolonged CPR/BMV (or when to continue prolonged CPR in the absence of MV).
**89. It would be helpful if there was a protocol with clear guidelines about for how long we should**	Critical need for developing clear protocol/policy for (when to stop)

**continue on CPR for such patients and when to stop.**	prolonged CPR/BMV (or when to continue prolonged CPR in the absence of MV).
**90. In my opinion if there was an official guideline from the Ministry of health that are liaised with Islamic affairs authorities, the decision for prolonged CPR continuation will change.**	Critical need for developing clear protocol/policy for (when to stop) prolonged CPR/BMV (or when to continue prolonged CPR in the absence of MV).
	Accommodating the religious and legal aspect of the issue in policy/decision making.
**91. More training (needed).**	Need for more/continuous training on CPR.
**92. PICU MUST BE AVILABLE AT ANY HOPITAL WITH STAFF TRAINIG.**	Post-resuscitation critical care support makes prolonged CPR futile.
**93. Training of all medical staff- all levels.**	Need for more/continuous training on CPR.
**94. We need to have a clear protocol.**	Critical need for developing clear protocol/policy for (when to stop) prolonged CPR/BMV (or when to continue prolonged CPR in the absence of MV).
**95. Sometimes there is a problem with providing suitable even bag and mask for respiratory support.**	Shortage/lack of essential medicines and equipment/resources to undertake prolonged CPR.
**96. Sometimes there is a problem with the availability of PICU beds.**	Post-resuscitation critical care support makes prolonged CPR futile.
**97. Making clear protocol.**	Critical need for developing clear protocol/policy for (when to stop) prolonged CPR/BMV (or when to continue prolonged CPR in the absence of MV).
**98. Legal staff in any hospital to share the decision.**	Accommodating the religious and legal aspect of the issue in policy/decision making.
**99. Call system to include the specialists in such care.**	Need for a dedicated resuscitation team in each hospital.
**100. We used to continue CPR awaiting bed availability in a tertiary care unit or PICU.**	Post-resuscitation critical care support makes prolonged CPR futile.
**101. Clear guidelines are very important in this situation.**	Critical need for developing clear protocol/policy for (when to stop) prolonged CPR/BMV (or when to

	continue prolonged CPR in the absence of MV).
**102. CPR Should not continue for more than 40 min.**	Prolonged CPR/BMV is futile.
**103. Juniors need clear teaching and guidance that they can prolong the resuscitation if they are looking for ventilator. Once there is no hope of finding a ventilator they should stop bagging.**	Prolonged CPR/BMV is futile.
	Need for mechanical ventilator (unavailability makes prolonged CPR futile).
**104. We hope to sign a clear protocol for this issue.**	Critical need for developing clear protocol/policy for (when to stop) prolonged CPR/BMV (or when to continue prolonged CPR in the absence of MV).
**105. Respiratory arrest if managed early there is good chance of saving the child.**	Other
**106. In Sudan we don't have clear guidelines or protocols when to stop prolonged CPR. we typically face such cases with no mechanical ventilation facilities. decisions usually are individual and depend on the acting team. in situations staff exhaustion can be one of causes to end such prolonged CPR. Ethical consideration is of the most causes to continue such events.**	Critical need for developing clear protocol/policy for (when to stop) prolonged CPR/BMV (or when to continue prolonged CPR in the absence of MV).
	Emotional toll/moral burden on doctor if CPR is stopped while heart is still beating.
	Decisions around prolonged CPR should be individualized.
**107. The issue of establishing a clear protocol is very important.**	Critical need for developing clear protocol/policy for (when to stop) prolonged CPR/BMV (or when to continue prolonged CPR in the absence of MV).
**108. Difficult to decide when to stop CPR.**	Difficult decision-making around prolonged CPR.

**Themes**
Critical need for developing clear protocol/policy for (when to stop) prolonged CPR/BMV (or when to continue prolonged CPR in the absence of MV).

Emotional toll/moral burden on doctor if CPR is stopped while heart is still beating.
Need for mechanical ventilator (unavailability makes prolonged CPR futile).
Lack of proper post-resuscitation critical care (need for pediatric intensive care setting)
Prolonged CPR/BMV is futile/has poor outcome.
Need for a dedicated resuscitation team in each hospital.
Need for more/continuous training on CPR.
Shortage/lack of human resources to undertake prolonged CPR.
Shortage/lack of essential medicines and equipment/resources to undertake prolonged CPR.
Prolonged CPR causes diversion of care from other patients.
Policy/decision making should accommodate religious and legal aspect.
Difficult decision-making around prolonged CPR.
Decisions around prolonged CPR should be/is individualized.
